# Silver nanoparticles are broad-spectrum bactericidal and virucidal compounds

**DOI:** 10.1186/1477-3155-9-30

**Published:** 2011-08-03

**Authors:** Humberto H Lara, Elsa N Garza-Treviño, Liliana Ixtepan-Turrent, Dinesh K Singh

**Affiliations:** 1 Department of Life Sciences, Winston-Salem State University, Winston Salem, NC 27110, USA; 2 Laboratorio de Terapia Celular, Departamento de Bioquimica y Medicina Molecular, Facultad de Medicina Universidad Autonoma de Nuevo Leon, Mexico

**Keywords:** Silver Nanoparticles, Virucides, Bactericides, HIV/AIDS, Antibacterial agents

## Abstract

The advance in nanotechnology has enabled us to utilize particles in the size of the nanoscale. This has created new therapeutic horizons, and in the case of silver, the currently available data only reveals the surface of the potential benefits and the wide range of applications. Interactions between viral biomolecules and silver nanoparticles suggest that the use of nanosystems may contribute importantly for the enhancement of current prevention of infection and antiviral therapies. Recently, it has been suggested that silver nanoparticles (AgNPs) bind with external membrane of lipid enveloped virus to prevent the infection. Nevertheless, the interaction of AgNPs with viruses is a largely unexplored field. AgNPs has been studied particularly on HIV where it was demonstrated the mechanism of antiviral action of the nanoparticles as well as the inhibition the transmission of HIV-1 infection in human cervix organ culture. This review discusses recent advances in the understanding of the biocidal mechanisms of action of silver Nanoparticles.

## Review

Historically, silver metal has been used widely across the civilizations for different purposes. Many societies use silver as jewelry, ornamentation and fine cutlery. Silver, jewelry, wares and cutlery was considered to impart health benefits to the users. In ancient Indian medical system (Ayurveda) silver has been described as therapeutic agent for many diseases. There is an increasing use of silver as an efficacious antibacterial and antifungal agent in wound care products and medical devices [1-4] including dental work and catheters [5-7]. Another application is to synthesize composites for use as water disinfecting filters [8]. Silver is also appearing more frequently in textiles, cosmetics [9], and even domestic appliances. It is worth mentioning some examples, such as inorganic composites with a slow silver release rate, which are currently used as preservatives in a variety of cosmetic products [10]; another current application includes new compounds of silica gel microspheres containing a silver thiosulfate complex, which are mixed into plastics for long-lasting antibacterial protection [11].

Metallic silver has also been used for surgical prosthesis and splints, fungicides, and coinage. Soluble silver compounds, such as silver salts, have been used for treating mental illness, epilepsy, nicotine addiction, gastroenteritis, stomatitis [12,13], and sexually transmitted diseases, including syphilis and gonorrhea [14]. Additionally, AgNO_3_, as eye drops, have been utilized to prevent gonococcal ophthalmic *neonatorum *in newborns by pediatricians for centuries [15]. Other agents derived from silver, such as silver sulfadiazine (AgSD) cream, have been used by surgeons, as topical treatments to heal burn wounds, for the past 60 years [16,17]. Utilizing these topical treatments, applied directly to the burn site, erythema decreased, while the expression of matrix metalloproteinases (MMPs) increased [18]. Recent advances in nanotechnology have enabled us to produce pure silver, as nanoparticles, which are more efficient than silver ions (AgSD and AgNO_3_) [19]. This has opened up whole new strategies to use pure silver against a wide array of pathogens, particularly multi-resistant pathogens which are hard to treat with available antibiotics. The biocidal activities of pure silver nanoparticles are discussed in subsequent sections of this review.

The multi-resistant pathogens due to antigenic shifts and/or drifts are ineffectively managed with current medications. This resistance to medication by pathogens has become a serious problem in public health; therefore, there is a strong need to develop new bactericides and virucides. Silver nanoparticles (AgNPs), having a long history of general use as an antiseptic and disinfectant, are able to interact with disulfide bonds of the glycoprotein/protein contents of microorganisms such as viruses, bacteria [20,21] and fungi [22]. Both silver nanoparticles and silver ions can change the three dimensional structure of proteins by interfering with S-S bonds and block the functional operations of the microorganism [23,24].

### Silver Nanoparticles

Nanoparticles are defined as particulate dispersions or solid particles with a size in the range of 10-100 nm [25]. AgNPs can be dissolved in a liquid environment that prevents their agglomeration or entrapped in a matrix that utilizes special drug carrier systems (e.g., the drug is dissolved, entrapped, encapsulated or attached to a nanoparticle matrix). These particles represent an interesting candidate for research as microbicides due to their effectiveness in small doses, minimal toxicity and side effects [26]. These attributes may contribute significantly to the enhancement of current prevention of infection and antiviral therapies [19,26].

**Figure 1:**
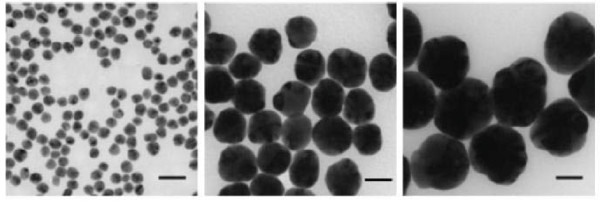
**Transmission electron microscopy (TEM) images of silver nanoparticles with diameters of 20 nm (Aldrich), 60 nm (Aldrich), and 100 nm (Aldrich), respectively**. Scale bars are 50 nm.

Particle size and size distribution are the most important characteristics of nanoparticle systems (Figure 1). They determine the *in vivo *distribution, biological fate, toxicity and the targeting ability of nanoparticle systems [27]. Available routes of administration include oral, nasal, parenteral or intra-ocular [28]. Despite these advantages, nanoparticles do have limitations. For example, their small size and large surface area can lead to particle-particle aggregation, making physical handling of nanoparticles difficult in liquid and dry forms [29]. This aggregation may lead to the loss of the properties associated with the nanoscale nature of the particles. The rate of agglomeration of nanoparticles is an important parameter for toxicology studies. Greulich *et al*. 2009 [30] reported that the agglomeration of AgNPs was specifically observed after the incubation of AgNPs in RPMI 1640 medium (a commonly used medium to dilute AgNPs) alone. However, washing AgNPs with RPMI 1640 medium containing Fetal Calf Serum (FCS) efficiently prevented agglomeration of AgNPs. Apart from agglomeration, particle sizes of AgNPs are also responsible for cytotoxicity. Yen *et al*. 2009 reported that smaller AgNPs (3 nm) are more cytotoxic than larger particles (25 nm) at a concentration of 10 μg/mL [31] signifying importance of particle size. Fukuoka and colleagues in an elegant experiment have demonstrated synthesis of necklace-shaped mono- and bimetallic nanowires for organic-inorganic hybrid mesoporous materials for better efficacy indicating not only the size of nanoparticle is important, but the shape and morphology are important as well [32]. Recent advances in Nanotechnology help in modulation of size and shape of nanoparticles and provide different ways of utilizing application of nanoparticles in diagnosis and treatment of various diseases. Using latest technology, Nanomaterials can also be tailored to facilitate their applications in other fields such as bioscience and medicine [3].

### AgNPs as Antibacterial Agents

AgNPs are attractive because they are non-toxic to the human body at low concentrations and have broad-spectrum antibacterial actions [33]. In fact, it is well known that Ag^+ ^ions and Ag-based compounds are toxic to microorganisms, possessing strong biocidal effects on at least 12 species of bacteria including multi-resistant bacteria like Methicillin-resistant *Staphylococcus aureus *(MRSA), as well as multidrug-resistant *Pseudomonas aeruginosa*, ampicillin-resistant E. coli O157:H7 and erythromycin-resistant S. pyogenes [2,4,21] suggesting that AgNPs are effective broadspectrum [34] biocides against a variety of drug-resistant bacteria, which makes them a potential candidate for use in pharmaceutical products and medical devices that may help to prevent the transmission of drug-resistant pathogens in different clinical environments [2,35]. Recently, Mecking and co-workers demonstrated that hybrids of silver nanoparticles with amphiphilic hyperbranched macromolecules exhibited effective antimicrobial surface coating agent properties [36].

The mechanism of the inhibitory effects of Ag^+ ^ions on microorganisms is not completely clear, however, AgNPs interact with a wide range of molecular processes within microorganisms resulting in a range of effects from inhibition of growth, loss of infectivity to cell death which depends on shape [37], size [31], concentration of AgNPs [38] and the sensitivity of the microbial species to silver [2,17,35,39-41]. Several studies have reported that the positive charge on the Ag^+ ^ion is crucial for its antimicrobial activity through the electrostatic attraction between the negatively charged cell membrane of the microorganism and the positively charged nanoparticles [1]. In contrast, Sondi and Salopek-Sondi reported that the antimicrobial activity of AgNPs on Gram-negative bacteria depends on the concentration of AgNPs and is closely associated with the formation of pits in the cell wall of bacteria [21]; consequently, AgNPs accumulated in the bacterial membrane disturbing the membrane permeability, resulting in cell death. However, because those studies included both positively charged Ag^+ ^ions and negatively charged AgNPs, this data is insufficient to explain the antimicrobial mechanism of positively charged silver nanoparticles. Therefore, we theorize that there is another possible mechanism. Amro *et al*. suggested that metal depletion may cause the formation of irregularly shaped pits in the outer membrane and change membrane permeability, which is caused by the progressive release of lipopolysaccharide molecules and membrane proteins [42]. Also, Sondi and Salopek-Sondi speculated that a similar mechanism may cause the degradation of the membrane structure of E. coli during treatment with AgNPs [21]. Although it is assumed that AgNPs are involved in some sort of binding mechanism, the mechanism of the interaction between AgNPs and components of the outer membrane is still unclear. Recently, Danilczuk and co-workers reported that Ag-generated free radicals derived from the surface of AgNPs were responsible for the antimicrobial activity [43]. However, Lara and colleagues in another report, proposed another mechanism of bactericidal action based on the inhibition of cell wall synthesis, protein synthesis mediated by the 30s ribosomal subunit, and nucleic acid synthesis [2]. The proteomic data revealed that a short exposure of *E. coli *cells to antibacterial concentrations of AgNPs resulted in an accumulation of envelope protein precursors, indicative of the dissipation of proton motive force [44]. Consistent with these proteomic findings, AgNPs were shown to destabilize the outer membrane, collapse the plasma membrane potential and deplete the levels of intracellular ATP [45].

The mode of action of AgNPs was also found to be similar to that of Ag^+ ^ions [45]; however, the effective concentrations of silver nanoparticles and Ag^+ ^ions were at nanomolar and micromolar levels. Therefore results in *E. coli *suggested silver nanoparticles may damage the structure of bacterial cell membrane and depress the activity of some membranous enzymes, which cause *E. coli *bacteria to die eventually [46].

### Silver Nanoparticles as Virucidal Agents

Virucidal agents differ from virustatic drugs in that they act directly and rapidly by lysing viral membranes on contact or by binding to virus coat proteins. Nevertheless, the interaction of AgNPs with viruses is still an unexplored field. However, the mechanism of action of AgNPs as an antiviral and virucidal has been studied against several enveloped viruses. Recently, it has been suggested that nanoparticles bind with a viral envelope glycoprotein and inhibit the virus by binding to the disulfide bond regions of the CD4 binding domain within the HIV-1 viral envelope glycoprotein gp120, as suggested by Elechiguerra and colleagues [47]. This fusion inhibition was later elegantly demonstrated by Lara and colleagues [19] in their latest report.

The antiviral effects of AgNPs on the hepatitis B virus (HBV) have been reported using a HepAD38 human hepatoma cell line. There has been evidence of high binding affinity of nanoparticles for HBV DNA and extracellular virions with different sizes (10 and 50 nm). Moreover, it has been demonstrated that AgNPs could also inhibit the production of HBV RNA and extracellular virions *in vitro*, which was determined using a UV-vs absorption titration assay. Further investigation will be needed to determine whether this binding activity prevents HBV virions from entering into host cells or not [39]. In an another report Sun and colleagues showed that AgNPs were superior to gold nanoparticles for cytoprotective activities toward HIV-1-infected Hut/CCR5 cells [48]. It is generally understood that Ag, in various forms, inactivates viruses by denaturing enzymes via reactions with sulfhydra, amino, carboxyl, phosphate, and imidazole groups [33,34,36,41,49]. However, it is necessary to design studies *in vivo *to increase therapeutic benefit and minimize adverse effects.

Among antiviral activities, the capacity of AgNPs to inhibit an influenza virus was determined in a MDCK cell culture and was demonstrated that with AgNPs at 0.5 μg/ml concentration viral infectivity was reduced. Nanosilver may interfere with the fusion of the viral membrane, inhibiting viral penetration into the host cell [40].

**Figure 2:**
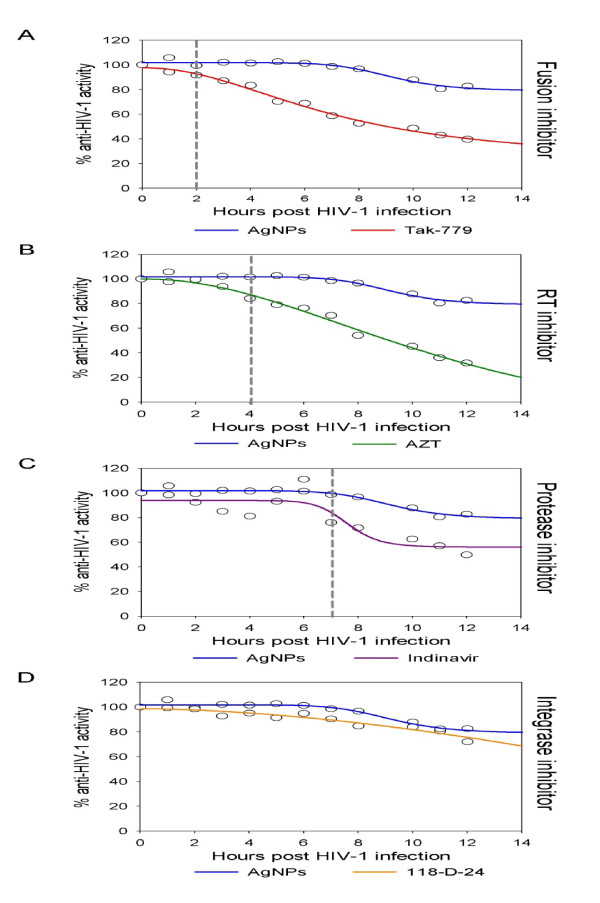
**Time-of-addition experiment**. HeLa-CD4-LTR-β-gal cells were infected with HIV-1_IIIB _and exposed to silver nanoparticles (1 mg/mL). Different antiretrovirals were added at different times post infection. Activity of silver nanoparticles was compared with (A) fusion inhibitor (Tak-779, 2 μM), (B) RT inhibitor (AZT, 20 μM), (C) protease inhibitor (Indinavir, 0.25 μM), and (D) integrase inhibitor (118-D-24, 100 μM). Dashed lines indicate the moment when the activity of the silver nanoparticles and the antiretroviral differ. The assays were performed in triplicate; the data points represent the mean and the colored lines are nonlinear regression curves performed with SigmaPlot 10.0 software. http://www.jnanobiotechnology.com/content/8/1/1/figure/F2

Lara and colleagues further demonstrated that AgNPs inhibited a variety of HIV-1 strains regardless of their tropism, clade and resistance to antiretrovirals [19]. The fact that AgNPs inhibited number of HIV-1 isolates suggest that their mode of action does not depend on cell tropism and that AgNPs are broad spectrum anti-HIV-1 agents. A cell-based fusion assay using Env expressing cells (HL2/3) and CD4 expressing cells mixture demonstrated that AgNPs efficiently blocked cell-cell fusion in a dose-dependent manner within the 1.0-2.5 mg/mL dose range including: Tak-779 (Fusion Inhibitor), AZT (NRTI), Indinavir (PI) and 118-D-24 (Integrase Inhibitor) as controls (Figure 2). In addition, efficient inhibitory activity of AgNPs against gp120-CD4 interaction was measured in a competitive gp120-capture ELISA. The results of the cell-based fusion assay confirm the hypothesis that AgNPs inhibit HIV-1 infection by blocking the viral entry, particularly the gp120-CD4 interaction [19]. Other studies also showed that AgNPs at non-toxic concentrations effectively inhibit arenavirus replication during the early phases of viral replication [50].

Continuing to assess antiviral mechanisms, a virus adsorption assay was performed to measure the inhibitory effects of AgNPs on virus adsorption to HeLa-CD4-LTR-β-gal cells, which were incubated with HIV_IIIB_, in the absence or presence of serial dilutions of AgNPs. After 2 h of incubation at 37°C, the cells were extensively washed with 1× PBS to remove the unadsorbed virus particles. Then the cells were incubated for 48 h, and the amount of viral infection was quantified with the Beta-Glo Assay System (Promega). The AgNPs inhibited the initial stages of the HIV-1 infection cycle of HIV_IIIB _virus to cells with an IC_50 _of 0.44 mg/mL.

Cell-free and cell-associated HIV-1 were pretreated at different concentrations of AgNPs, and were centrifuged and washed to separate the virus from the AgNPs and then infect the indicator cells. The cell-associated virus includes infected cells that transmit the infection by fusing with non-infected target cells. In addition, AgNPs treatment of chronically infected H9+ cells as well as human PBMC+ (cell-associated HIV) resulted in decreased infectivity in a dose-dependent manner [19].

Time-of-addition experiments (TAE) for HIV revealed that silver nanoparticles have other sites of intervention on the viral life cycle besides fusion or entry (Figure 2). This could be explained by silver nanoparticles suppressing the expression of TNF-α, a cytokine that plays a critical role in HIV-1 pathogenesis, by incrementing HIV-1 transcription. The inhibition of the TNF-α activated transcription might also be a target for the anti-HIV activity of silver nanoparticles. Having a variety of targets in the HIV-1 replication cycle makes silver nanoparticles agents that are not prone to contribute to the emergence of resistant strains [26].

### The Antiviral Effect of AgNPs as a Topical Agent on Human Cervical Tissue

**Figure 3:**
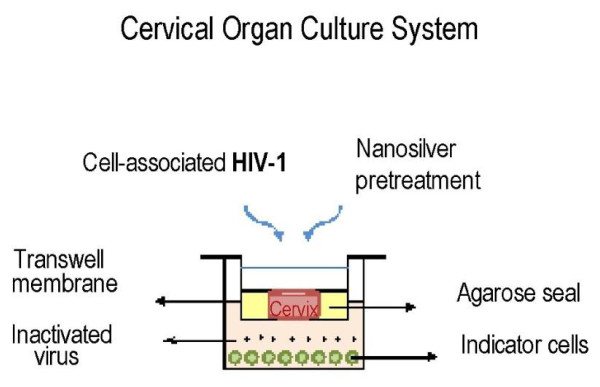
**Human cervical culture model**. a) To rule out possible leaks in the agarose seal, Dextran blue was added to the upper chamber on day 6 of the culture. Its presence in the lower chamber was determined 20 h later to all Transwells used in the experiments, along with the negative control well with agarose only, b) the other negative control alone, with tissue and virus but without treatment or challenge and c) positive control well with tissue alone, infected with only the HIV-1 virus. d) Inhibition of HIV-1 transmission; the cervical tissue was treated with PVP-coated AgNPs at different concentrations in a Replens gel or RPMI + 10% FCS media, which was then infected with HIV-1_IIIB_. HIV transmission or inhibition of transmission across the mucosa was determined in the lower chamber by formation of syncytia using indicator cells (MT-2). http://www.jnanobiotechnology.com/content/8/1/15/figure/F3

**Figure 4:**
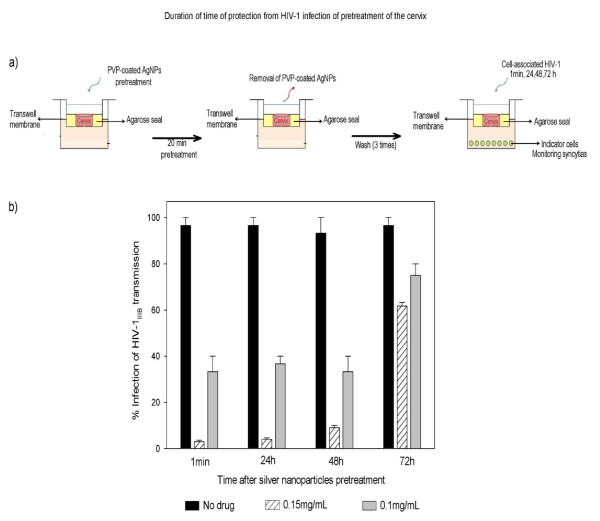
**Protection from HIV-1 infection following pre-treatment of the cervical explant with PVP-coated AgNPs**. a) Cervical explants were exposed to 0.1 or 0.15 mg/mL PVP-coated AgNPs in RPMI + 10% FCS media for 20 minutes. After thoroughly washing extracellular PVP-coated AgNPs from the cervical explant, and after 1 minute, 24 h, 48 h and 72 h, cell-free virus (HIV-1_IIIB_) [(5 × 10^5 ^TCID_50_)] was added to the upper chamber. To verify the neutralization of HIV-1 transmission, we cultured the indicator cells (MT-2) in the lower chamber and evaluated the inhibition of the HIV-1 infection. b) Cervical explants were exposed to HIV-1 in the absence of PVP-coated AgNPs as a control and to 0.1 or 0.15 mg/mL of PVP-coated AgNPs as pretreatment. Graphs show values of the means ± standard deviations from three separate experiments. Graphs were created using the SigmaPlot 10.0 software. http://www.jnanobiotechnology.com/content/8/1/15/figure/F5

In an experiment evaluating AgNPs application on Human cervical tissue as an anti-HIV-1 agent, Lara and colleagues [26] found that AgNPs provided protection against the transmission of cell-free and cell-associated HIV-1. They had used an excellent human cervical tissue culture model to elucidate anti-HIV-1 activity of AgNPs within one minute after the topical treatment on the human cervical tissue (Figure 3). The similar effect was found for 20 minutes time point of topical pretreatment and washing of the AgNPs. The human cervical tissue culture remained protected against infection with HIV-1 for as long as 48 h, demonstrating a long-lasting tissue protection afforded by AgNPs. This lasting protection is necessary for a topical vaginal microbicide to ensure safety against infection even for many hours after gel application and, even more importantly, after the gel is washed away (Figure 4) [26].

### AgNPs as Topical Agents in Mucosal Human Tissue

Recent studies showed that pre-treatment of human cervical tissues with AgNPs increased the proliferation of lymphocytes, presumably due to activation of the immune cells [26,14,51,52]. The increased proliferation of lymphocytes also increases inflammatory process in situ by contributing in wound healing *in vivo *[53]. The development of inflammatory process in cervical tissue helps activation of innate defenses against invading microbes. These changes during inflammation in cervical tissue are chiefly regulated under hormonal conditions by estradiol and progesterone [54,55]. Further studies are necessary to evaluate topical use of nanoparticles applied repeatedly to record chronic response, toxicity (i.e., genetic, reproductive, and carcinogenic toxicities) and long-term side effects, susceptibility to opportunistic infections or significant changes in tissue architecture. Studies should also be performed to evaluate occurrence of any hypersensitivity/photosensitivity and AgNPs effect on condom integrity before AgNPs can be included in a topical gel for human use [56,57].

### AgNPs cytotoxicity

The AgNPs have been shown to be cytotoxic at higher concentration than 6 μg/mL. Hsin and colleagues provided evidence for the molecular mechanism of AgNPs induction of cytotoxicity. They showed that AgNPs acted through ROS and JNK to induce apoptosis via the mitochondrial pathway in NIH3T3 fibroblast cells [58]. Park and colleagues reported cytotoxicity using silver nanoparticles prepared by dispersing them in fetal bovine serum, as a biocompatible material, on a cultured macrophage cell line, which induced cellular apoptosis [59]. Furthermore, AgNPs decreased intracellular glutathione levels, increased NO secretion, increased TNF-α protein and gene levels, and increased the gene expression of matrix metalloproteinases, such as MMP-3, MMP-11, and MMP-19. Kim and colleagues demonstrated cytotoxicity induced by AgNPs in human hepatoma HepG2 cells and observed that AgNPs agglomerated in the cytoplasm and nuclei of treated cells, and induced intracellular oxidative stress, independent of the toxicity of the Ag^+ ^ions [1]. In a similar study, Kawata and colleagues showed an upregulation of DNA repair-associated genes in hepatoma cells cultured with low dose AgNPs, suggesting possible DNA damaging effects [60,61]. Recent studies demonstrated that uptake of AgNPs occurs mainly through clathrin mediated endocytosis and macropinocytosis [38], however it seems that AgNPs have multiples cellular targets that vary among different cell types.

## Conclusions

The emergence and spread of antibiotic resistance pathogen is an alarming concern in clinical practice. Many organisms such as MRSA, HIV-1, Hepatitis-B Virus, and Ampicillin resistant *E.coli *are difficult to treat. There is a need of a cheap broad-active agent that can be used against variety of pathogen. The AgNPs have been found to be effective against many viruses and bacterial species. The use of noble metals at nanosizes to treat many conditions is gaining importance. The recent development in nanotechnology has provided tremendous impetus in this direction due to its capacity of modulating metals into nanosizes and various shapes, which drastically changes the chemical, physical and optical properties and their use. The efficacy of AgNPs against HIV-1 has been reported by many laboratories including ours [19,26]. It has been shown that AgNPs have got anti-HIV-1 activity and can help the host immune system against HIV-1. This has laid ground for the development of new, potent antiviral drugs capable of preventing HIV infection and controlling virus replication. Recently, it has been demonstrated that AgNPs function as broad-spectrum virucidal and bactericidal agents, and in addition, increase wound healing. Nonetheless, conclusive safety has not been demonstrated extensively in animal models, and therefore, additional testing of AgNPs is needed before they can be used in clinical applications.

## List of Abbreviations Used

AgNPs: Silvernanoparticles; HIV-1: Human Immunodeficiency Virus-1; AgNO_3_: Silver Nitrate; AgSD: Silver Sulfadiazine; MMPs: Matrix Metalloproteinases; S-S Bonds: Disulfide Bonds; FCS: Fetal Calf Serum; MRSA: Methicillin-resitant *Staphylococcus aureus*; ATP: Adenosine triphosphate; HBV: Hepatitis B Virus; MDCK: Madin-Darby Canine Kidney cells; CD4: Cluster Differentiation-4; AZT: Azidothymidine; PBMC: Peripheral Blood Mononuclear cells; TAE: Time of Addition Experiments; TNF-α: Tumor Necrosis Factor-Alpha; IC_50_: The Half Maximal Inhibitory Concentration; ROS: Reactive Oxygen Species; JNK: **c-**Jun N-terminal Kinase; NO: Nitric Oxide.

## Competing interests

The authors declare that they have no competing interests.

## Authors' contributions

All authors read and approved the final manuscript. HHL participated in AgNPs cytotoxicity, AgNPs as topical agents in mucosal human tissue, and overall design of this review article. ENGT participated in AgNPs as antibacterial agent portion of this review, and overall design of this review article along with HHL. LIT participated in antiviral effect of AgNPs as a topical agent on Human cervical tissue. DKS participated in AgNPs as virucidal agents, and editing and revision of this report. His lab provided materials and resources used in this study.

## Authors Information

DKS: is an associate professor of microbiology at the Winston Salem State University. DKS' lab is working on development of a DNA vaccine for HIV/AIDS. His other research interest involves prevention of HIV-1 transmission at the cervical/vaginal mucosal surfaces. His current research is funded by two NIH grants.

## References

[B1] Kim JS Kuk E Yu KN Kim JH Park SJ Lee HJ Kim SH Park YK Park YH Hwang CY Kim YK Lee YS Jeong DH Cho MH Antimicrobial effects of silver nanoparticles Nanomedicine 2007 3 95 101 17379174 10.1016/j.nano.2006.12.001

[B2] Lara HH Ayala-Nuñez NV Ixtepan-Turrent L Rodriguez-Padilla C Bactericidal effect of silver nanoparticles against multidrug-resistant bacteria World Journal of Microbiology and Biotechnology 2010 26 615 621 10.1007/s11274-009-0211-3

[B3] Salata O Applications of nanoparticles in biology and medicine J Nanobiotechnology 2004 2 3 10.1186/1477-3155-2-3 15119954 PMC419715

[B4] Shahverdi AR Fakhimi A Shahverdi HR Minaian S Synthesis and effect of silver nanoparticles on the antibacterial activity of different antibiotics against Staphylococcus aureus and Escherichia coli Nanomedicine 2007 3 168 171 17468052 10.1016/j.nano.2007.02.001

[B5] Catauro M Raucci MG De GF Marotta A Antibacterial and bioactive silver-containing Na2O × CaO × 2SiO2 glass prepared by sol-gel method J Mater Sci Mater Med 2004 15 831 837 15387420 10.1023/b:jmsm.0000032825.51052.00

[B6] Crabtree JH Burchette RJ Siddiqi RA Huen IT Hadnott LL Fishman A The efficacy of silver-ion implanted catheters in reducing peritoneal dialysis-related infections Perit Dial Int 2003 23 368 374 12968845

[B7] Khare MD Bukhari SS Swann A Spiers P McLaren I Myers J Reduction of catheter-related colonisation by the use of a silver zeolite-impregnated central vascular catheter in adult critical care J Infect 2007 54 146 150 10.1016/j.jinf.2006.03.002 16678904

[B8] Jain P Pradeep T Potential of silver nanoparticle-coated polyurethane foam as an antibacterial water filter Biotechnol Bioeng 2005 90 59 63 10.1002/bit.20368 15723325

[B9] Lansdown AB A pharmacological and toxicological profile of silver as an antimicrobial agent in medical devices Adv Pharmacol Sci 2010 2010 910686 21188244 10.1155/2010/910686 PMC3003978

[B10] Kokura S Handa O Takagi T Ishikawa T Naito Y Yoshikawa T Silver nanoparticles as a safe preservative for use in cosmetics Nanomedicine 2010 6 570 574 20060498 10.1016/j.nano.2009.12.002

[B11] Morones JR Elechiguerra JL Camacho A Holt K Kouri JB Ramirez JT Yacaman MJ The bactericidal effect of silver nanoparticles Nanotechnology 2005 16 2346 2353 10.1088/0957-4484/16/10/059 20818017

[B12] Alidaee MR Taheri A Mansoori P Ghodsi SZ Silver nitrate cautery in aphthous stomatitis: a randomized controlled trial Br J Dermatol 2005 153 521 525 10.1111/j.1365-2133.2005.06490.x 16120136

[B13] Tanweer F Hanif J Re: Silver nitrate cauterisation, does concentration matter? Clin Otolaryngol 2008 33 503 504 18983401 10.1111/j.1749-4486.2008.01803.x

[B14] Gougeon ML Lecoeur H Dulioust A Enouf MG Crouvoiser M Goujard C Debord T Montagnier L Programmed cell death in peripheral lymphocytes from HIV-infected persons: increased susceptibility to apoptosis of CD4 and CD8 T cells correlates with lymphocyte activation and with disease progression J Immunol 1996 156 3509 3520 8617980

[B15] Hoyme UB Clinical significance of Crede's prophylaxis in germany at present Infect Dis Obstet Gynecol 1993 1 32 36 10.1155/S1064744993000080 18476203 PMC2364679

[B16] George N Faoagali J Muller M Silvazine (silver sulfadiazine and chlorhexidine) activity against 200 clinical isolates Burns 1997 23 493 495 10.1016/S0305-4179(97)00047-8 9429028

[B17] Illingworth B Bianco RW Weisberg S In vivo efficacy of silver-coated fabric against Staphylococcus epidermidis J Heart Valve Dis 2000 9 135 141 10678386

[B18] Hoffmann S Silver sulfadiazine: an antibacterial agent for topical use in burns. A review of the literature Scand J Plast Reconstr Surg 1984 18 119 126 10.3109/02844318409057413 6377481

[B19] Lara HH Ayala-Nunez NV Ixtepan-Turrent L Rodriguez-Padilla C Mode of antiviral action of silver nanoparticles against HIV-1 J Nanobiotechnology 2010 8 1 10.1186/1477-3155-8-1 20145735 PMC2818642

[B20] Furr JR Russell AD Turner TD Andrews A Antibacterial activity of Actisorb Plus, Actisorb and silver nitrate J Hosp Infect 1994 27 201 208 10.1016/0195-6701(94)90128-7 7963461

[B21] Sondi I Salopek-Sondi B Silver nanoparticles as antimicrobial agent: a case study on E. coli as a model for Gram-negative bacteria J Colloid Interface Sci 2004 275 177 182 10.1016/j.jcis.2004.02.012 15158396

[B22] Gajbhiye M Kesharwani J Ingle A Gade A Rai M Fungus-mediated synthesis of silver nanoparticles and their activity against pathogenic fungi in combination with fluconazole Nanomedicine 2009 5 382 386 19616127 10.1016/j.nano.2009.06.005

[B23] Chung YC Chen IH Chen CJ The surface modification of silver nanoparticles by phosphoryl disulfides for improved biocompatibility and intracellular uptake Biomaterials 2008 29 1807 1816 10.1016/j.biomaterials.2007.12.032 18242693

[B24] Liau SY Read DC Pugh WJ Furr JR Russell AD Interaction of silver nitrate with readily identifiable groups: relationship to the antibacterial action of silver ions Lett Appl Microbiol 1997 25 279 283 10.1046/j.1472-765X.1997.00219.x 9351278

[B25] Zhang G Niu A Peng S Jiang M Tu Y Li M Wu C Formation of novel polymeric nanoparticles Acc Chem Res 2001 34 249 256 10.1021/ar000011x 11263883

[B26] Lara HH Ixtepan-Turrent L Garza-Trevino EN Rodriguez-Padilla C PVP-coated silver nanoparticles block the transmission of cell-free and cell-associated HIV-1 in human cervical culture J Nanobiotechnology 2010 8 15 10.1186/1477-3155-8-15 20626911 PMC2911397

[B27] Panyam J Labhasetwar V Biodegradable nanoparticles for drug and gene delivery to cells and tissue Adv Drug Deliv Rev 2003 55 329 347 10.1016/S0169-409X(02)00228-4 12628320

[B28] Mohanraj VJ Chen Y (Eds) Nanoparticles J Pharmaceutical Research 2006 5 561 573

[B29] Kondow T Mafune F Structures and dynamics of molecules on liquid beam surfaces Annu Rev Phys Chem 2000 51 731 761 10.1146/annurev.physchem.51.1.731 11031298

[B30] Greulich C Kittler S Epple M Muhr G Koller M Studies on the biocompatibility and the interaction of silver nanoparticles with human mesenchymal stem cells (hMSCs) Langenbecks Arch Surg 2009 394 495 502 10.1007/s00423-009-0472-1 19280220

[B31] Yen HJ Hsu SH Tsai CL Cytotoxicity and immunological response of gold and silver nanoparticles of different sizes Small 2009 5 1553 1561 10.1002/smll.200900126 19326357

[B32] Fukuoka A Sakamoto Y Guan S Inagaki S Sugimoto N Fukushima Y Hirahara K Iijima S Ichikawa M Novel templating synthesis of necklace-shaped mono- and bimetallic nanowires in hybrid organic-inorganic mesoporous material J Am Chem Soc 2001 123 3373 3374 10.1021/ja004067y 11457076

[B33] Baker C Pradhan A Pakstis L Pochan DJ Shah SI Synthesis and antibacterial properties of silver nanoparticles J Nanosci Nanotechnol 2005 5 244 249 10.1166/jnn.2005.034 15853142

[B34] Rai M Yadav A Gade A Silver nanoparticles as a new generation of antimicrobials Biotechnol Adv 2009 27 76 83 10.1016/j.biotechadv.2008.09.002 18854209

[B35] Yamanaka M Hara K Kudo J Bactericidal actions of a silver ion solution on Escherichia coli, studied by energy-filtering transmission electron microscopy and proteomic analysis Appl Environ Microbiol 2005 71 7589 7593 10.1128/AEM.71.11.7589-7593.2005 16269810 PMC1287701

[B36] Aymonier C Schlotterbeck U Antonietti L Zacharias P Thomann R Tiller JC Mecking S Hybrids of silver nanoparticles with amphiphilic hyperbranched macromolecules exhibiting antimicrobial properties Chem Commun (Camb) 2002 3018 3019 10.1039/b208575e 12536795

[B37] Pal S Tak YK Song JM Does the antibacterial activity of silver nanoparticles depend on the shape of the nanoparticle? A study of the Gram-negative bacterium Escherichia coli Appl Environ Microbiol 2007 73 1712 1720 10.1128/AEM.02218-06 17261510 PMC1828795

[B38] Asharani PV Hande MP Valiyaveettil S Anti-proliferative activity of silver nanoparticles BMC Cell Biol 2009 10 65 10.1186/1471-2121-10-65 19761582 PMC2759918

[B39] Lu L Sun RW Chen R Hui CK Ho CM Luk JM Lau GK Che CM Silver nanoparticles inhibit hepatitis B virus replication Antivir Ther 2008 13 253 262 18505176

[B40] Mehrbod P Motamed N Tabatabaian M Soleimani Estyar R Amini E Shahidi M In Vitro Antiviral Effect of "Nanosilver" on Influenza Virus DARU 2009 17 88 93

[B41] Ruparelia JP Chatterjee AK Duttagupta SP Mukherji S Strain specificity in antimicrobial activity of silver and copper nanoparticles Acta Biomater 2008 4 707 716 10.1016/j.actbio.2007.11.006 18248860

[B42] Amro NA Kotra LP Wadu-Mesthrige K Bulychev A Mobashery S Liu G (Eds) High-resolution atomic force microscopy studies of the Escherichia coli outer membrane: structural basis for permeability Langmuir 2000 16 2789 2796 10.1021/la991013x

[B43] Danilczuk M Lund A Sadlo J Yamada H Michalik J Conduction electron spin resonance of small silver particles Spectrochim Acta A Mol Biomol Spectrosc 2006 63 189 191 10.1016/j.saa.2005.05.002 15978868

[B44] Lok CN Ho CM Chen R He QY Yu WY Sun H Tam PK Chiu JF Che CM Proteomic analysis of the mode of antibacterial action of silver nanoparticles J Proteome Res 2006 5 916 924 10.1021/pr0504079 16602699

[B45] Dibrov P Dzioba J Gosink KK Hase CC Chemiosmotic mechanism of antimicrobial activity of Ag(+) in Vibrio cholerae Antimicrob Agents Chemother 2002 46 2668 2670 10.1128/AAC.46.8.2668-2670.2002 12121953 PMC127333

[B46] Li WR Xie XB Shi QS Zeng HY Ou-Yang YS Chen YB Antibacterial activity and mechanism of silver nanoparticles on Escherichia coli Appl Microbiol Biotechnol 2010 85 1115 1122 10.1007/s00253-009-2159-5 19669753

[B47] Elechiguerra JL Burt JL Morones JR Camacho-Bragado A Gao X Lara HH Yacaman MJ Interaction of silver nanoparticles with HIV-1 J Nanobiotechnology 2005 3 6 10.1186/1477-3155-3-6 15987516 PMC1190212

[B48] Sun RW Chen R Chung NP Ho CM Lin CL Che CM Silver nanoparticles fabricated in Hepes buffer exhibit cytoprotective activities toward HIV-1 infected cells Chem Commun (Camb) 2005 5059 5061 10.1039/b510984a 16220170

[B49] Borkow G Gabbay J Putting copper into action: copper-impregnated products with potent biocidal activities FASEB J 2004 18 1728 1730 15345689 10.1096/fj.04-2029fje

[B50] Speshock JL Murdock RC Braydich-Stolle LK Schrand AM Hussain SM Interaction of silver nanoparticles with Tacaribe virus J Nanobiotechnology 2010 8 19 10.1186/1477-3155-8-19 20718972 PMC2936366

[B51] Poon VK Burd A In vitro cytotoxity of silver: implication for clinical wound care Burns 2004 30 140 147 10.1016/j.burns.2003.09.030 15019121

[B52] Wright JB Lam K Buret AG Olson ME Burrell RE Early healing events in a porcine model of contaminated wounds: effects of nanocrystalline silver on matrix metalloproteinases, cell apoptosis, and healing Wound Repair Regen 2002 10 141 151 10.1046/j.1524-475X.2002.10308.x 12100375

[B53] Tian J Wong KK Ho CM Lok CN Yu WY Che CM Chiu JF Tam PK Topical delivery of silver nanoparticles promotes wound healing ChemMedChem 2007 2 129 136 10.1002/cmdc.200600171 17075952

[B54] Fahey JV Wright JA Shen L Smith JM Ghosh M Rossoll RM Wira CR Estradiol selectively regulates innate immune function by polarized human uterine epithelial cells in culture Mucosal Immunol 2008 1 317 325 10.1038/mi.2008.20 19079193 PMC4815904

[B55] Wira CR Fahey JV The innate immune system: gatekeeper to the female reproductive tract Immunology 2004 111 13 15 10.1111/j.1365-2567.2004.01796.x 14678193 PMC1782397

[B56] Cremel M Berlier W Hamzeh H Cognasse F Lawrence P Genin C Bernengo JC Lambert C Dieu-Nosjean MC Delézay O Characterization of CCL20 secretion by human epithelial vaginal cells: involvement in Langerhans cell precursor attraction J Leukoc Biol 2005 78 158 166 10.1189/jlb.0305147 15831560

[B57] McGowan I Microbicides: a new frontier in HIV prevention Biologicals 2006 34 241 255 10.1016/j.biologicals.2006.08.002 17097303

[B58] Hsin YH Chen CF Huang S Shih TS Lai PS Chueh PJ The apoptotic effect of nanosilver is mediated by a ROS- and JNK-dependent mechanism involving the mitochondrial pathway in NIH3T3 cells Toxicol Lett 2008 179 130 139 10.1016/j.toxlet.2008.04.015 18547751

[B59] Park EJ Yi J Kim Y Choi K Park K Silver nanoparticles induce cytotoxicity by a Trojan-horse type mechanism Toxicol In Vitro 2010 24 872 878 10.1016/j.tiv.2009.12.001 19969064

[B60] Kawata K Osawa M Okabe S In vitro toxicity of silver nanoparticles at noncytotoxic doses to HepG2 human hepatoma cells Environ Sci Technol 2009 43 6046 6051 10.1021/es900754q 19731716

[B61] Miura N Shinohara Y Cytotoxic effect and apoptosis induction by silver nanoparticles in HeLa cells Biochem Biophys Res Commun 2009 390 733 737 10.1016/j.bbrc.2009.10.039 19836347

